# *FTO* and *PLAG1* Genes Expression and *FTO* Methylation Predict Changes in Circulating Levels of Adipokines and Gastrointestinal Peptides in Children

**DOI:** 10.3390/nu13103585

**Published:** 2021-10-13

**Authors:** Wojciech Czogała, Wojciech Strojny, Magdalena Schab, Agnieszka Grabowska, Karol Miklusiak, Wojciech Kowalczyk, Agnieszka Łazarczyk, Przemysław Tomasik, Szymon Skoczeń

**Affiliations:** 1 Department of Pediatric Oncology and Hematology, University Children’s Hospital of Krakow, 30-663 Krakow, Poland; czogala@tlen.pl (W.C.); wojciech.strojny@mp.pl (W.S.); magdalenaschab@interia.pl (M.S.); 2 Department of Pediatric Oncology and Hematology, Faculty of Medicine, Jagiellonian University Medical College, 30-663 Krakow, Poland; 3 Department of Medical Genetics, Faculty of Medicine, Jagiellonian University Medical College, 30-663 Krakow, Poland; aga.grabowska@uj.edu.pl; 4 Student Scientific Group of Pediatric Oncology and Hematology, Jagiellonian University Medical College, 30-663 Krakow, Poland; karolmiklusiak@gmail.com (K.M.); w.kowalczyk@student.uj.edu.pl (W.K.); agnieszka.lazarczyk@student.uj.edu.pl (A.Ł.); 5 Department of Clinical Biochemistry, Faculty of Medicine, Jagiellonian University Medical College, 30-663 Krakow, Poland; p.tomasik@uj.edu.pl

**Keywords:** epigenetics, expression, *FTO*, *PLAG1*, children, adipokines, gastrointestinal tract hormones

## Abstract

Adipokines and gastrointestinal tract hormones are important metabolic parameters, and both epigenetic factors and differential gene expression patterns may be associated with the alterations in their concentrations in children. The function of the *FTO* gene (*FTO* alpha-ketoglutarate dependent dioxygenase) in the regulation of the global metabolic rate is well described, whereas the influence of protooncogene *PLAG1* (*PLAG1* zinc finger) is still not fully understood. A cross-sectional study on a group of 26 children with various BMI values (15.3–41.7; median 28) was carried out. The aim was to evaluate the dependencies between the level of methylation and expression of aforementioned genes with the concentration of selected gastrointestinal tract hormones and adipokines in children. Expression and methylation were measured in peripheral blood mononuclear DNA by a microarray technique and a restriction enzyme method, respectively. All peptide concentrations were determined using the enzyme immunoassay method. The expression level of both *FTO* and *PLAG1* genes was statistically significantly related to the concentration of adipokines: negatively for apelin and leptin receptor, and positively for leptin. Furthermore, both *FTO* methylation and expression negatively correlated with the concentration of resistin and visfatin. Cholecystokinin was negatively correlated, whereas fibroblast growth factor 21 positively correlated with methylation and expression of the *FTO* gene, while *FTO* and *PLAG1* expression was negatively associated with the level of cholecystokinin and glucagon-like peptide-1. The *PLAG1* gene expression predicts an increase in leptin and decrease in ghrelin levels. Our results indicate that the *FTO* gene correlates with the concentration of hormones produced by the adipose tissue and gastrointestinal tract, and *PLAG1* gene may be involved in adiposity pathogenesis. However, the exact molecular mechanisms still need to be clarified.

## 1. Introduction

Adipokines and gastrointestinal peptides, secreted by fat tissue and enteroendocrine cells, respectively, are crucial factors regulating food intake, as well as glucose and lipid homeostasis. Consequently, they are strongly associated with both the development and implications of obesity—a condition currently becoming one of the major concerns in pediatrics. According to the WHO (World Health Organization) data, in 2016, the prevalence of obesity in the 5–19 age group was equal to 6.8% globally, 21.4% in the USA, and 9.1% in Poland [1,2]. Both epigenetic factors and differential gene expression patterns have been previously reported to be associated with the alterations in the concentrations of adipokines and gastrointestinal hormones [3,4,5,6,7,8,9,10,11]. In turn, such alterations can be considered to contribute to the development of obesity, to result from it, or to be associated with changes in BMI (body mass index) without known causality. Out of the hormones studied in this paper, those which are typically upregulated in obesity include leptin [12,13], visfatin [14,15], apelin [16] and FGF21 (fibroblast growth factor 21) [17] (although, in case of the last two, there are conflicting data regarding childhood adiposity [16,18,19,20,21,22]), whereas adiponectin [23,24,25], ghrelin [13,26] and, probably, GLP-1 (glucagon-like peptide-1) [27] are usually downregulated. In contrast, levels of resistin [28,29,30] and cholecystokinin (CCK) [31] do not seem to differ between obese and lean individuals.

The *FTO* gene (*FTO* alpha-ketoglutarate dependent dioxygenase) [32,33] has been extensively described as linked to obesity, both in adults and in children, as indicated by SNP (single nucleotide polymorphism) research [34,35,36], as well as epigenome-wide association studies [37,38] (according to EWAS Atlas [39]). Furthermore, there is a growing body of evidence for the existing connection between *FTO* and concentrations of certain adipokines and gastrointestinal peptides. Namely, *FTO* polymorphisms have been shown to be related to altered levels of leptin, adiponectin, apelin, resistin, visfatin, ghrelin and GLP-1 [40,41,42,43,44,45,46,47,48].

The protooncogene *PLAG1* (*PLAG1* zinc finger) [33,49] is associated with neoplasms such as lipoblastoma or pleomorphic adenoma of the salivary gland [50,51]. In addition, it is currently being explored as a novel gene connected with anthropometric parameters [52,53,54]. So far, there have been no studies investigating its relationship with the concentrations of gastrointestinal hormones. However, *PLAG1* cord blood methylation, possibly affecting its expression, has been recently linked to neonatal levels of leptin [3].

In our previous study, we showed that the expression of the *FTO* gene is elevated in childhood obesity and, along with the methylation of this gene, predicts changes in certain parameters of glucose-lipid metabolism, thus further emphasizing the role of this gene in adiposity [55]. We also identified an association between the *PLAG1* expression and body fat content, showing an involvement of this gene in determining body composition. Here, we aimed to further explore the role of these genes in the pathogenesis of childhood obesity and its complications by analyzing the correlation between their expression and circulating levels of leptin, soluble leptin receptor, adiponectin, apelin, resistin, visfatin, ghrelin, cholecystokinin, FGF21 and GLP-1. To adjust for the possible influence of confounders on the observed associations, we constructed a multiple linear regression model. Furthermore, to investigate the epigenetic background for changes in the levels of studied hormones, we analyzed them in the context of *FTO* gene methylation.

## 2. Materials and Methods

In total, 26 children (16 boys, 10 girls) aged 6.6–17.7 (median: 14.65) years were included in the analysis of the cross-sectional study. The inclusion criteria for the study were children aged below 18 years, and the absence of acute and chronic diseases, except for primary obesity. The mean BMI percentile of the group was 82.47 ± 30.02.

### 2.1. Microarray Analysis

Blood samples (1.5 mL) were collected from each child. Total RNA extraction from blood mononuclears was performed using the RiboPure Blood Kit (Ambion, Life Technologies, Carlsbad, CA, USA). Whole-genome expression was assessed using the GeneChip Human Gene 1.0 ST Arrays (Affymetrix, Santa Clara, CA, USA). All procedures were done following the manufacturer’s instructions (GeneChip Whole Transcript sense Target Labeling Assay Manual, Version 4, Affymetrix, Santa Clara, CA, USA). Despite the fact that the goal of the study was to investigate only two genes, expression was analyzed on a genome-wide level for the purpose of other research.

### 2.2. Methylation Analysis

#### 2.2.1. DNA and Selection of Fragments

Genomic DNA was isolated from the peripheral blood using MasterPure DNA Purification Kit for Blood (Epicentre). *FTO* (Chr16) methylation (upstream, 16:53703684-53703899) was tested with the method based on methylation-dependent (MDRE) and methylation-sensitive (MSRE) restriction enzymes. MDREs detect regions of DNA that are highly methylated, oppositely to MSREs, which can target the regions with low degrees of methylation [56,57]. The regions of interest in both genes were established according to the methyl-DIP data available in the Ensembl database [58] (Supplementary Materials). The palindrome fragments containing CCGG targets for the MDRE and MSRE enzymes were used. Table 1 contains the sequences of each primer that was used in the qPCR. The primers were designed using PrimerQuest (Integrated DNA Technologies) [59]. For this, we evaluated the methylation level of *PLAG1* gene, region 1 ((*PLAG1* exon1) 8:56211059-56211208). We used the *PLAG1* methylation to control for *FTO*. The genome reference sequence used was GRCh38.p13.

#### 2.2.2. Restriction Enzyme Digestion

For MSRE reaction, we used the HpaII enzyme in combination with MspI (EpiJET, Thermo). These enzymes are isoschizomers that target CCGG sequences. Methylated CpG sites are resistant to HpaII digestion but still prone to MspI cleavage. Digestions with both enzymes were carried out overnight at 37 °C and terminated at 90 °C for 10 min. Both methylated and unmethylated pUC19/SmaI DNA were used to determine the digestion ratio. A total of 50 ng and 100 ng of genomic DNA was cleaved by FspE (MDRE) and HpaII (MSRE), respectively. FspEI modification-dependent endonuclease was used as the MDRE (New England BioLabs, Ipswich, MA, USA). The reaction was carried out in 30 µL in the presence of 2.5 U of enzyme and reaction buffer supplemented with Enzyme Activator Solution and BSA. After 4 h of incubation at 37 °C, the reaction was stopped at 80 °C for 20 min.

#### 2.2.3. qPCR

Real-time experiments were performed according to the MIQE guidelines. For all reactions, we applied intercalating dye chemistry with SYBR Green, as well as hot start iTaq DNA polymerase (iTaq Universal SYBR Green Supermix, Bio-Rad). All reactions were run in triplicate on the CFX384 Touch Real-Time PCR Detection System (Bio-Rad). On each reaction plate, serial dilutions of the control DNA were run to establish the PCR efficiency and correct calculations.

#### 2.2.4. Methylation Data Processing and Statistical Analysis

All downstream data processing and statistical analyses were performed with the statistical software R [60] together with the methylumi and limma [61] packages of the Bioconductor project [62].

Moderated t-statistics for each contrast and probe were created using an empirical Bayes model as implemented in limma (eBayes command). The *p*-values were adjusted for multiple comparisons as proposed by Benjamini and Hochberg [63], and an adjusted *p*-value > 0.05 was considered nonsignificant (ns).

### 2.3. Study Protocol

Blood concentrations of CCK, ghrelin, GLP-1, adiponectin, apelin, leptin, leptin receptor, resistin and visfatin were measured at fasting, as well as at 60 and 120 min of the standard oral glucose tolerance test (OGTT) conducted using 1.75 g of anhydrous glucose per kg of body mass (maximum of 75 g). Both tubes that contained EDTA or aprotinin (BectonDickinson, Plymouth, UK) and tubes without anticoagulant were used in the blood sample collection process. The blood samples were immediately transported to the diagnostic laboratory at a temperature of +4 °C and centrifuged for 15 min at a relative centrifugal force of 1590× *g*.

### 2.4. Laboratory Measurements

We assessed the concentrations of various hormones by the EIA method: CCK, ghrelin, GLP-1 (Phoenix Pharmaceuticals, Inc., Burlingame, CA, USA) and FGF-21 (Millipore Corporation, Burlington, MA, USA). Plasma samples for adipokine and soluble leptin receptor analyses were stored at −80 °C until the time of the measurement. Plasma concentrations of the peptides were measured using the following assays: enzyme immunoassay (EIA) (Phoenix Pharmaceuticals, Burlingame, CA, USA)—adiponectin, apelin and visfatin; enzyme-amplified sensitivity immunoassay (Biosource; Nivelles, Belgium)—leptin; (EIA) (BioVendor Research and Diagnostic Products, Brno, Czech Republic)—leptin receptor and resistin.

### 2.5. Anthropometric Measurements

Body weight and height were measured to the nearest 0.1 kg and 0.1 cm, respectively, using a balanced scale and a stadiometer. All measurements were conducted by an anthropometrist. Online World Health Organization (WHO) BMI calculators were used to determine the body mass index (BMI), BMI percentile (BMI_Perc) and BMI SD [64].

### 2.6. Statistical Analysis

The interval data are presented as mean ± SD, while the categorical data are presented as frequencies (N) and proportions (%). If any data were missing, the case was not included in the analysis for the given variable. All area-under-the-curve (AUC) values were calculated by applying the trapezoidal rule. The Spearman’s correlation coefficient (r) was used to estimate the relationship between the interval variables.

Multivariate linear regression models incorporating the expression and the methylation of genes were constructed in attempt to identify a link between the *FTO* gene methylation, *FTO* gene expression and *PLAG1* gene expression with the serum level of adipokines and gastrointestinal tract hormones. A standard threshold of *p*-value = 0.05 was used to determine data significance. The Benjamini–Hochberg (BH) procedure was used to correct for multiple testing (assuming FDR = 0.05), and an adjusted p^BH^ < 0.05 was considered as significant. All analyzes were performed with Statistica 13.3 software (Statsoft Inc., Tulsa, OK, USA).

The study protocol gained the approval of the Permanent Ethical Committee for Clinical Studies of the Medical College of the Jagiellonian University (KBET/249/B/2013 26 October 2013). All parents, children and adults signed a written informed consent before blood sample collection. That study respects the statements of The Code of Ethics of the World Medical Association (Declaration of Helsinki), printed in the British Medical Journal (18 July 1964).

## 3. Results

The characteristics of the study groups are presented in Table 2. Our study included 16 boys and 10 girls (61.54%/38.46%). The mean age was 13.98 ± 2.6 years. The mean values of the anthropometric parameters were height: 164.85 ± 13.9 cm, weight: 76.62 ± 24.98 kg, BMI: 27.87 ± 7.48 kg/m^2^, BMI percentiles: 82.47 ± 30.02 and BMI SD: 4.35 ± 3.9. The percentage of the methylation level of the *FTO* gene was 4.35 ± 3.9%, while the methylation level of the *PLAG1* gene was 0.8 ± 0.8%. However, for this region of *PLAG1* gene, there was a large loss of data due to the low digestion rate of the samples. As a result, we did not include the studied parameter in further analysis. Mean *FTO* expression was 266.72 ± 87.46, while *PLAG1* expression was 82 ± 21.73. The exact concentrations of the adipokines and gastrointestinal hormones, as well as the values of the methylation and expression of genes, are given in Table 3 and Table S1.

### 3.1. Spearman’s Correlation Results of Adipokines with FTO Gene Methylation, Expression and PLAG1 Gene Expression

In the case of the *FTO* gene methylation level, Spearman′s correlation coefficient r was insignificant.

By analyzing the *FTO* expression, a statistically significant correlation was obtained for apelin (AUC apelin r = −0.758, p^BH^ < 0.001), leptin (AUC leptin r = 0.713, p^BH^ < 0.001), leptin receptor (leptin receptor r = −0.622, p^BH^ = 0.005) and resistin (resistin -OGTT 60 min r = −0.507, p^BH^ = 0.020). Detailed information on the investigated parameters is presented in Table 4 and Table S2. 

According to our observations, the *PLAG1* expression was associated with the serum concentration of apelin (AUC apelin r = −0.579, p^BH^ = 0.014), leptin (AUC leptin r = 0.676, p^BH^ = 0.002) and soluble leptin receptor (AUC leptin receptor r = −0.488, p^PH^ = 0.04). Detailed data are presented in Table 4.

### 3.2. Spearman’s Correlation Results of Gastrointestinal Tract Hormones with FTO Gene Methylation, Expression and PLAG1 Gene Expression

The level of *FTO* gene methylation correlated with the plasma concentration of cholecystokinin (AUC CCK r = −0.508, p^BH^ = 0.044) and FGF21 (AUC FGF r = 0.556, p^BH^ = 0.04). In the case of the *FTO* gene expression level, a correlation was demonstrated for the plasma levels of cholecystokinin (AUC CCK r = −0.613, p^BH^ = 0.044), FGF21 (AUC FGF21 r = 0.525, p^BH^ = 0.016) and GLP-1 (AUC GLP-1 r = −0.638, p^BH^ = 0.003). The level of *PLAG1* expression did not correlate with gastrointestinal tract hormones serum levels. Detailed information on these dependencies is presented in Table 5 and Table S3.

### 3.3. Multiple Linear Regression Model Explaining the Effect of FTO Gene Methylation, FTO Gene Expression and PLAG1 Gene Expression on the Plasma Concentrations of Adipokines and Gastrointestinal Tract Hormones

To assess the impact of the *FTO* gene methylation, *FTO* gene expression and *PLAG1* gene expression on the plasma adipokines and gastrointestinal tract hormones, a linear regression model was created to investigate these relationships (additionally considering the sex of the subjects). In such a model, the *FTO* gene methylation level showed an effect on the AUC of CCK (B = −0.25 ± 0.12, *p* = 0.05), AUC of FGF21 (B = 20.51 ± 8.31, *p* = 0.023) and AUC of ghrelin (B = −39.47 ± 16.94, *p* = 0.032). *FTO* expression, on the other hand, affected the AUC of apelin (B = −1.09 ± 0.51, *p* = 0.045), AUC of adiponectin (B = 1.94 ± 0.80, *p* = 0.028), AUC of GLP-1 (B = −0.25 ± 0.1, *p* = 0.020) and AUC of leptin receptor (B = −4.00 ± 1.83, *p* = 0.041). The *PLAG1* expression affected the AUC of ghrelin (B = −99.6 ± 33.3, *p* = 0.008) and AUC of leptin (B = 10.44 ± 4.12, *p* = 0.020). The exact results of the tested model are shown in Table 6.

## 4. Discussion

**Figure 1:**
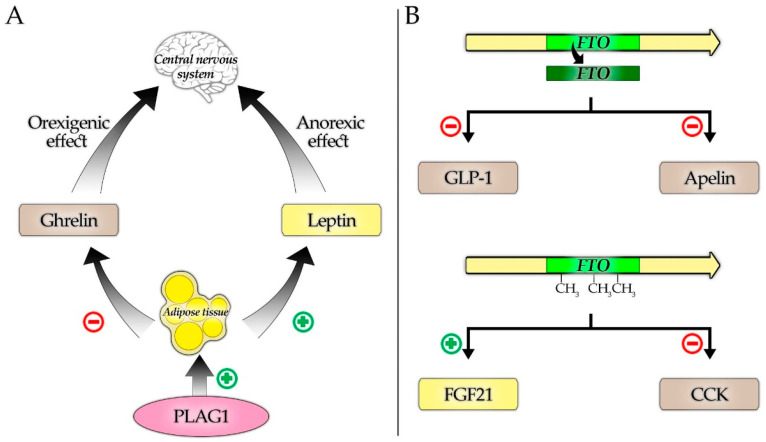
Suggested mechanisms of interactions between the studied genes and hormones. (**A**) Regulation of ghrelin and leptin, key appetite and satiety controlling hormones by *PLAG1*, secondary to its fat tissue stimulating properties. (**B**) Selected hormones downregulated in response to the increased *FTO* expression (upper part) and influenced by the higher *FTO* methylation (down part).

In our previous research, we demonstrated differences in the methylation and expression of the *FTO* gene between healthy and obese children [55]. In the current study, we showed that the expression and methylation level of the *FTO* gene and the expression of the *PLAG1* gene are related to the concentrations of selected adipokines and gastrointestinal tract hormones. Finally, we conducted a multiple linear regression analysis to predict the changes in lipid metabolism parameters in children based on the *FTO* and *PLAG1* genes. Our key findings, together with the hypothetical interactions explaining them, are illustrated in Figure 1.

### 4.1. FTO Gene

The *FTO* Alpha-Ketoglutarate Dependent Dioxygenase, based on the GeneCards—The Human Genes Database [65,66,67], belongs to a nuclear protein of the AlkB-related non-haem iron and 2-oxoglutarate-dependent oxygenase superfamily. Its exact physiological function is unknown. However, it has strong association with body mass index, obesity risk and type 2 diabetes (DM2) [67]. It regulates adipogenesis and body fat accumulation, thereby contributing to the regulation of body size and weight [68,69,70,71,72]. These findings suggest the existence of a relationship between the *FTO* gene and adipokines and gastrointestinal tract hormones. However, the expression and methylation of the *FTO* gene have not been described yet. In our previous study, we found that the obese children showed significantly higher expression of the *FTO* gene in comparison to the healthy controls, as well as significantly higher methylation values for the *FTO* gene [55], which confirmed a considerable role of the *FTO* gene in the development of obesity.

In our study, we showed that higher level of both *FTO* gene methylation and expression in children was associated with a decreased serum concentrations of adipokines and gastrointestinal tract hormones during fasting and after the oral glucose tolerance test, although the obtained data was not always statistically significant. Only two hormones—leptin and FGF-21—correlated positively with the expression and methylation of the *FTO* gene, and these results were statistically significant. Thus, it seems that the *FTO* gene may be an important factor regulating the mechanism of changes in the concentration of adipokines and gastrointestinal tract hormones in children. Namely, we hypothesize that higher expression of this gene causes an inhibition of the production or secretion of tissue hormones, and, as a consequence, a decrease in their circulating levels.

#### 4.1.1. *FTO* and Adipokines

We showed a statistically significant relationship between the *FTO* gene expression and the concentrations of apelin, leptin, leptin receptor and resistin, while the relationship with the adiponectin and visfatin concentrations were not significant. In case of the *FTO* gene methylation, its correlation with the visfatin concentration was statistically significant, both during fasting and after OGTT, as was the association with the concentration of resistin, but only after the OGTT test. However, after the Benjamini-Hochberg adjustment, these results lost their statistical significance, similar to the remaining results. 

Leptin, as one of the only adipokines analyzed in our study, correlated positively with the expression of the *FTO* gene. Leptin is a main protein regulating energy homeostasis in the body, inhibiting feeding and promoting energy expenditure. Moreover, mutation in its gene causes severe or morbid obesity with hypogonadism in humans [66,67]. The leptin receptor, in turn, is a receptor for leptin, and is involved in the regulation of fat metabolism [66,67]. In contrast to leptin, its concentration correlated negatively with the *FTO* gene expression. Leptin is among factors which are the most potently upregulated in response to fat tissue accumulation, and the *FTO* expression is higher in obese children. Combined, those two facts may explain the observed association, despite the supposed inhibitory action of *FTO* on the production or secretion of adipokines. The found opposite correlations of *FTO* gene expression with leptin and the leptin receptor could be explained by the downregulation mechanism caused by the higher leptin concentration, causing the saturation of its receptor and, consequently, a decrease in its availability. The relationship of the rs9939609 *FTO* gene variant with the highest leptin concentration has been well described independently of potential confounders, including in subjects with adiposity [40,41,42,73].

The relationship between the *FTO* gene and the concentration of apelin is very interesting. In our study, apelin correlated negatively with the *FTO* expression, both during fasting and after OGGT. Apelin is responsible for, i.a., cardiovascular function and insulin secretion, and its functional disorders are associated with DM2 and cardiovascular system diseases [66,67]. Most of the current data indicate that apelin levels increase with increasing BMI [22,74]. However, these data refer to prepubertal children or adults. In children in adolescence, the situation looks different, and in pubertal obese children, the apelin level may be lower compared to non-obese children [21]. Another factor that causes an increase in the apelin levels is physical activity. After an intense 12-week exercise period, people with DM2 experienced a significant increase in plasma apelin levels [75]. Son et al. showed that there is a positive correlation between plasma apelin concentration and lean body mass after intense physical activity [76]. The concentration of apelin is also significantly higher in people with an active lifestyle compared to a sedentary lifestyle [77]. Our observations showed that the increase in the *FTO* expression may be one of the predictors of the apelin serum level, which may have a beneficial effect on the lipid profile and carbohydrate metabolism of patients. It is worth noting that the concentration of apelin in adolescents may act differently than in people of a different age. Furthermore, additional factors, such as lifestyle or physical exercise, have an influence on the concentration of apelin.

The data concerning the *FTO* gene and both resistin and visfatin levels are inconsistent. Resistin is a hormone potentially linking obesity to DM2 [66,67]. We obtained statistically significant data for resistin correlated with the expression of the *FTO* gene, but only after the OGTT test. Thus, it is likely that the fasting resistin concentration is not related to the *FTO* gene, but that the resistin release as a result of loading with a carbohydrate meal is associated with the *FTO* gene. Visfatin is involved as a protein in many important biological processes, including metabolism. Serum visfatin was nearly significantly negatively correlated with the *FTO* expression at 60 min of OGTT and in the case of the AUC parameter. The lack of unambiguous data on changes in the concentrations of visfatin and resistin in adolescents with different body weight may indicate the existence of a more direct relationship and regulatory mechanism between the *FTO* gene and concentrations of both of the hormones.

Adiponectin is involved in the control of fat metabolism and insulin sensitivity, with direct anti-diabetic, anti-atherogenic and anti-inflammatory activities. It is also associated with disorders of the adipose tissue and insulin metabolism [66,67]. In our studies, adiponectin did not show a significant correlation with the *FTO* gene. However, all the correlations we obtained with the *FTO* gene were negative. The reason for poor correlation in our study may be attributed to the small patient group. In contrast, the study of the rs9939609 variant by Duicu et al. was performed on a group which consisted of about 400 children [42]. Further research into the *FTO* gene and adiponectin concentration is necessary.

#### 4.1.2. *FTO* and Gastrointestinal Tract Hormones

The *FTO* gene is strongly associated with body mass index, obesity risk, the regulation of fat mass, body size, body fat accumulation and body weight [66,67]. Therefore, its interaction with gastrointestinal hormones, responsible for the feeling of hunger and satiety and food intake control, may play a key role in understanding the mechanisms leading to obesity. In our study, three of the four tested gastrointestinal tract hormones (CCK, GLP-1 and ghrelin) were negatively correlated with *FTO* gene. Only FGF21 correlated positively. The results were statistically significant during fasting and after the OGTT test for both the methylation and expression of *FTO* gene for CCK and FGF21, and in the case of GLP-1 for *FTO* expression only.

We obtained the negative correlation between the concentration of cholecystokinin and *FTO* gene methylation, as well as the *FTO* expression, at every timepoint we studied. These results may be explained by the fact that cholecystokinin induces satiety by interacting with the CCK-1 receptors located in specialized regions of the hindbrain [78]. We showed that higher expression of the *FTO* gene occurs usually in people with higher BMI, in whom the process of activating the satiety center may be disturbed. Therefore, this association may result from the pre-existing eating disorders. It is also supported by the fact that CCK is the main protein in the regulation of energy intake in the organism [79]. However, it is worth mentioning that, in our study, in the multiple linear regression analysis for the *FTO* gene, the methylation of the *FTO* gene was the best predictor of CCK plasma concentrations among all studied gastrointestinal tract hormones in children.

Glucagon-like peptide 1 is a peptide hormone formed in the intestinal epithelial endocrine L-cells in the differential processing of proglucagon. Its main action is to stimulate insulin secretion and to inhibit glucagon secretion, thereby limiting postprandial glucose excursions [80]. We found a strong negative correlation between the *FTO* gene expression and GLP-1 concentration both during fasting and after the OGTT test. It is possible that, in addition to genetic factors, including the *FTO* gene, environmental factors and physical activity have a significant impact on GLP-1 concentration. However, this be studied as the subject of further research.

In the case of ghrelin, the negative correlation we found was not statistically significant. Ghrelin is a powerful appetite stimulant, which plays an important role in energy homeostasis. It is secreted when the stomach is empty, and then binds to the growth hormone secretagogue receptor in the hypothalamus, causing causes the secretion of the growth hormone [66,67]. These facts may suggest that ghrelin could be related to the *FTO* gene, but the available data are contradictory. Magno et al., studying the *FTO* rs9939609 polymorphism, showed that the subject with AA genotype indeed had decreased postprandial ghrelin concentration [46]. In contrast, Danaher et al. did not confirm the relationship between the rs9939609 polymorphism of the *FTO* gene and the preprandial or postprandial ghrelin level [81]. These various reports may highlight the complexity of the interaction mechanisms between *FTO* and ghrelin, but more research is needed to fully understand them.

FGF21 is a protein that stimulates the uptake of glucose in adipose tissue. An FGF21 disturbance may cause, i.a., acquired lipodystrophy [66,67]. It was the only gastrointestinal tract hormone for which we showed the positive correlation with both *FTO* gene expression and *FTO* methylation. The association was observed during fasting and after the OGTT test, as well as for the AUC parameter. The genome-wide meta-analysis by Tanaka et al. showed that both the *FTO* and FGF21 genes are very strongly connected to food intake regulation [82]. Therefore, it appears that both genes may well reflect the degree of eating disorder in children, and perhaps even predict this condition, acting synergistically.

### 4.2. PLAG1 Gene

The *PLAG1* (*PLAG1* zinc finger) is a protooncogene encoding a protein with two putative nuclear localization signals, which functions as a transcription factor. It is responsible for the upregulation of genes such as *IGF2, CRABP2, CRLF1, PIGF* and *CRIP2*. The *PLAG1* function triggers cellular proliferation and is associated with the development of lipoblastomas and pleomorphic adenomas of the salivary gland [50,51,66,83]. It is also commonly overexpressed in AML (acute myeloid leukemia) and hepatoblastoma [84,85]. In addition, studies have indicated that *PLAG1* could contribute to the determination of body composition. According to the GWAS Catalog [86,87], *PLAG1* polymorphisms are associated with traits such as birth weight [52], lean body mass [53] and BMI-adjusted hip circumference [54]. In our previous research, we showed that *PLAG1* expression correlated positively with body fat percent in children [55]. The possible involvement of *PLAG1* in fat tissue growth suggests that it could also influence the amount of synthesized adipokines. Similarly, the concentrations of gastrointestinal tract hormones, which regulate food intake, thereby also affecting body composition, could be associated with *PLAG1* function.

#### 4.2.1. *PLAG1* and Adipokines

In our study, we showed that the *PLAG1* expression correlated with levels of certain adipokines measured during OGTT—positively in the case of leptin, and negatively in the case of the leptin receptor and apelin. However, the multiple linear regression analysis revealed that an association independent of confounders occurred only in case of leptin concentration. There were no statistically significant outcomes regarding the concentrations of adiponectin, resistin and visfatin.

Kadakia et al. found that higher cord blood methylation of the *PLAG1* gene, probably resulting in the inhibition of its expression, is associated with lower leptin levels [3]. Our results further confirm the likely existence of a link between these two factors. Concentrations of IGF-2, the key *PLAG1* target gene, were shown to be higher in children with obesity compared to the control group [88], and were found to be associated with increased leptin levels in adult, normal-weight patients [89]. However, Kleiman et al. showed that leptin expression is inhibited, rather than promoted, by IGF-2 [90]. These conflicting results suggest that a pathway linking *PLAG1* to leptin could be independent of IGF-2. However, it remains unclear whether there is a direct mechanism connecting *PLAG1* to leptin synthesis on a molecular level. Therefore, the most likely reason for the found association is the aforementioned increase in the fat tissue content related to *PLAG1* expression, which ultimately results in augmented leptin production (Figure 1). The aforementioned mechanism of leptin receptor being downregulated by the increased concentration of its ligand could also explain the found negative correlation between the *PLAG1* expression and leptin receptor levels.

An observed negative correlation between the *PLAG1* expression and apelin concentration was not confirmed by multiple linear regression analysis. We suppose that this correlation is unlikely to arise from a simple interaction between the *PLAG1* and apelin systems, but rather results from one of the possible confounders which could alter apelin levels, such as low physical activity [77]. Furthermore, Reinehr et al. showed that apelin concentrations in children did not vary depending on BMI or body fat percent [20], which could explain why *PLAG1* expression does not cause an increase in apelin production despite being associated with higher fat tissue content.

#### 4.2.2. *PLAG1* and Gastrointestinal Tract Hormones

The construction of multiple linear regression model revealed a negative association between the *PLAG1* expression and concentrations of ghrelin. The levels of CCK, GLP-1 and FGF21 did not correlate significantly with the expression of *PLAG1*.

Ghrelin concentrations are typically decreased in obesity, except for Prader–Willi syndrome, where elevated ghrelin secretion is considered one of the factors leading to overeating [13,26]. The obesity-associated reduction in levels of ghrelin, which is an orexigenic factor, could be considered a compensatory mechanism aimed to prevent further weight gain [91]. A negative correlation between the body fat percent and ghrelin levels, which was reported in a study by Tschöp et al. [26], provides a very clear explanation of our obtained results given the known association of *PLAG1* and fat tissue content (Figure 1). Reduced ghrelin concentration in adiposity most likely results from the decreased sensitivity of ghrelin-producing cells in the stomach to noradrenalin, which normally stimulates ghrelin secretion, or from the increased abundance of duodenal somatostatin cells, resulting in an augmented somatostatin-mediated suppression of ghrelin cells [13,91]. The exact mechanism linking increased fat content and, therefore, *PLAG1*, to the described processes leading to decreased ghrelin levels is yet to be elucidated.

### 4.3. Limitations

The study population of 26 participants yields a relatively small cohort, which could possibly undermine the validity of the outcomes. Future studies using larger samples are required for definite confirmation of the obtained results.

Out of the two studied genes, the analysis included the methylation data only for the *FTO* gene. The *PLAG1* methylation was not included in the calculations due to the fact that the CpG sites within this gene, which we initially intended to investigate, proved to be of no influence on the expression of the gene [55].

Although analyzing gene expression in peripheral blood mononuclear cells in nutrigenomic research is supported by studies [92,93,94], there is a risk that the outcomes could be affected by variations in the proportions of cell types comprising the mononuclear cells. Similarly, the methylation results could be influenced by differences in the proportions of the cell types in the whole blood.

## 5. Conclusions

Higher expression and, in selected cases, methylation of the *FTO* gene, lowers the levels of adipokines and gastrointestinal tract hormones in children. This effect is especially clear for apelin, resistin, leptin receptor, cholecystokinin and GLP-1. In contrast, the serum levels of leptin and FGF21 increase in the context of increasing the *FTO* expression. All these factors are crucial in weight gain and obesity development.

We also showed that the *PLAG1* gene expression predicts an increase in leptin and decrease in ghrelin levels, with both hormones being key regulators of appetite and satiety. *PLAG1* may be involved in certain aspects of obesity pathogenesis. However, its role in the development of childhood adiposity may be more complex.

This is the first study linking the adipokines and gastrointestinal hormones concentrations with the expression of *PLAG1* and *FTO* genes. To confirm the results and to discover the exact molecular mechanisms of the found associations, further research including larger populations is necessary.

## Figures and Tables

**Table 1:** Sequences of primers used for the qPCR reaction.

Gene	Localization	Fragment Size	No. of CCGG Sites	Forward	Reverse
*FTO*	upstream	216bp	2	CAACTCCAGGGCCTTCTC	GGAGCCTGCCATGTTTCT
*PLAG1*	exon1	202bp	2	ACAATGGCTGCTGGAAAGA	CCCTGATATTTCTCCCGCTAAA

**Table 2:** Characteristics of the study group. Values are presented as mean ± standard deviation.

Baseline Characteristics	Study Group *N* = 26
boys/girls *n* (%)	16/10 (61.54%/38.46%)
Age (years)	13.98 ± 2.6
Height (cm)	164.85 ± 13.9
Weight (kg)	76.62 ± 24.98
BMI (kg/m^2^)	27.87 ± 7.48
BMI (percentile)	82.47 ± 30.02
BMI SD	2.05 ± 1.64

**Table 3:** The metabolic parameters of the study group. Values are presented as mean ± standard deviation.

Baseline Characteristics	Study Group *N* = 26
*FTO* gene methylation (%)	4.35 ± 3.9
*FTO* expression	266.72 ± 87.46
*PLAG1* gene methylation (%)	0.08 ± 0.08
*PLAG1* expression	82 ± 21.73
AUC Adiponectin (µg/mL/h)	7.01 ± 4.48
AUC apelin (ng/mL/h)	4.96 ± 3.47
AUC leptin (ng/mL/h)	55.08 ± 49.81
AUC leptin receptor (ng/mL/h)	21.44 ± 13.7
AUC resistin (ng/mL/h)	7.47 ± 1.94
AUC visfatin	21.86 ± 10.83
AUC FGF21 (ng/mL/h)	264.75 ± 157.18
AUC Cholecystokinin (ng/mL/h)	4.61 ± 2.72
AUC ghrelin (ng/mL/h)	955.42 ± 302.77
AUC GLP-1 (ng/mL/h)	1.89 ± 0.82

**Table 4:** Correlation results of the adipokines serum levels with the *FTO* gene methylation, expression and *PLAG1* gene expression.

Adipokines	*FTO* Gene Methylation		*FTO* Expression		*PLAG1* Expression	
	Spearman’s Correlation Coefficient r	*p*/p^BH^ Value	Spearman’s Correlation Coefficient r	*p*/p^BH^ Value	Spearman’s Correlation Coefficient r	*p*/p^BH^ Value
AUC adiponectin (µg/mL/h)	−0.29	0.203/0.271	−0.221	0.336/0.384	0.007	0.975/0.975
AUC apelin (ng/mL/h)	−0.388	0.067/0.322	−0.758	<0.001/<0.001	−0.579	0.004/0.014
AUC leptin (ng/mL/h)	0.294	0.164/0.262	0.713	<0.001/<0.001	0.676	<0.001/0.002
AUC leptin receptor (ng/mL/h)	−0.176	0.422/0.440	−0.622	0.002/0.005	−0.493	0.017/0.045
AUC resistin (ng/mL/h)	−0.356	0.088/0.302	−0.434	0.034/0.054	−0.19	0.373/0.471
AUC visfatin (ng/mL/h)	−0.364	0.105/0.252	−0.442	0.045/0.064	−0.263	0.249/0.374

**Table 5:** Correlation results of the gastrointestinal tract hormones serum levels with the *FTO* gene methylation, expression and *PLAG1* gene expression.

Gastrointestinal Tract Hormones	*FTO* Gene Methylation		*FTO* Expression		*PLAG1* Expression	
	Spearman’s Correlation Coefficient r	*p*/p^BH^ Value	Spearman’s Correlation Coefficient r	*p*/p^BH^ Value	Spearman’s Correlation Coefficient r	*p*/p^BH^ Value
AUC CCK (ng/mL/h)	−0.508	0.011/0.044	−0.613	0.001/0.008	−0.489	0.015/0.12
AUC FGF21 (ng/mL/h)	0.556	0.005/0.04	0.525	0.008/0.016	0.236	0.266/0.304
AUC Ghrelin (ng/mL/h)	−0.377	0.076/0.122	−0.265	0.222/0.254	−0.446	0.033/0.106
AUC GLP-1 (ng/mL/h)	−0.371	0.081/0.118	−0.638	0.001/0.003	−0.457	0.029/0.155

**Table 6:** Multiple linear regression analysis of the *FTO* gene methylation, *FTO* gene expression and *PLAG1* expression as predictors of changes in the plasma concentrations of adipokines and gastrointestinal tract hormones in children. Only the non-standardized regression coefficients ^1^ with *p* < 0.05 are shown.

Hormone	Gene (Non-Standardized Regression Coefficient (B) ± SEM, *p*-Value)	*R* ^2^_adj_	*p*/p^BH^-Value for *R*^2^
AUC apelin (ng/mL/h)	*FTO* expression (−1.09 ± 0.51, 0.045)	0.402	**0.009/0.018**
AUC CCK (ng/mL/h)	*FTO* methylation (−0.25 ± 0.12, 0.05)	0.435	**0.002/0.008**
AUC FGF21 (ng/mL/h)	*FTO* methylation (20.51 ± 8.31, 0.023)	0.297	**0.029/0.046**
AUC ghrelin (ng/mL/h)	*FTO* methylation (−39.47 ± 16.94, 0.032) *PLAG1* expression (−99.6 ± 33.3, 0.008)	0.260	**0.049**/0.056
AUC GLP-1 (ng/mL/h)	*FTO* expression (−0.25 ± 0.1, 0.020)	0.364	**0.004/0.011**
AUC leptin (ng/mL/h)	*PLAG1* expression (10.44 ± 4.12, 0.020)	0.537	<**0.001/0.006**
AUC leptin receptor (ng/mL/h)	*FTO* expression (−4.00 ± 1.83, 0.041)	0.239	**0.042**/0.056
AUC adiponectin (µg/mL/h)	*FTO* expression (−1.94 ± 0.80, 0.028)	0.142	0.098/0.098

^1^ Non-standardized regression coefficients were calculated per increment of *FTO* gene methylation, *FTO* gene expression and *PLAG1* gene expression. The increments for presented genes were chosen based on the gene baseline gene expression and *FTO* methylation for each gene, and are as follows: *FTO* methylation-1, *FTO* expression-50, *PLAG1* expression-10. Models with *p*-Value < 0.05 for *R*^2^*_adj_* are bolded.

## Data Availability

The datasets generated for this study are available on request to the corresponding author.

## Supplementary Materials

The following are available online at https://www.mdpi.com/article/10.3390/nu13103585/s1, Methylation regions, Table S1: The metabolic parameters of the study group (including values at OGTT time points), Table S2: Correlation results of adipokines serum levels with *FTO* gene methylation, expression and *PLAG1* gene expression (including values at OGTT time points), Table S3: Correlation results of gastrointestinal tract hormones serum levels with *FTO* gene methylation, expression and *PLAG1* gene expression (including values at OGTT time points).

## References

[1] Overweight/Obesity. https://www.who.int/data/gho/data/themes/topics/indicator-groups/indicator-group-details/GHO/overweight-obesity.

[2] Bentham J., Di Cesare M., Bilano V., Bixby H., Zhou B., Stevens G.A., Riley L.M., Taddei C., Hajifathalian K., Lu Y. (2017). Worldwide Trends in Body-Mass Index, Underweight, Overweight, and Obesity from 1975 to 2016: A Pooled Analysis of 2416 Population-Based Measurement Studies in 128·9 Million Children, Adolescents, and Adults. Lancet.

[3] Kadakia R., Zheng Y., Zhang Z., Zhang W., Josefson J.L., Hou L. (2019). Association of Cord Blood Methylation with Neonatal Leptin: An Epigenome Wide Association Study. PLoS ONE.

[4] Aslibekyan S., Do A.N., Xu H., Li S., Irvin M.R., Zhi D., Tiwari H.K., Absher D.M., Shuldiner A.R., Zhang T. (2017). CPT1A Methylation Is Associated with Plasma Adiponectin. Nutr. Metab. Cardiovasc. Dis..

[5] Nakatochi M., Ichihara S., Yamamoto K., Ohnaka K., Kato Y., Yokota S., Hirashiki A., Naruse K., Asano H., Izawa H. (2015). Epigenome-Wide Association Study Suggests That SNPs in the Promoter Region of RETN Influence Plasma Resistin Level via Effects on DNA Methylation at Neighbouring Sites. Diabetologia.

[6] Mishra A., Kohli S., Dua S., Thinlas T., Mohammad G., Pasha M.A.Q. (2015). Genetic Differences and Aberrant Methylation in the Apelin System Predict the Risk of High-Altitude Pulmonary Edema. Proc. Natl. Acad. Sci. USA.

[7] Wiemerslage L., Islam R., Van Der Kamp C., Cao H., Olivo G., Ence-Eriksson F., Castillo S., Larsen A.L., Bandstein M., Dahlberg L.S. (2017). A DNA Methylation Site within the KLF13 Gene Is Associated with Orexigenic Processes Based on Neural Responses and Ghrelin Levels. Int. J. Obes..

[8] Li P., Gao X., Sun X., Li W., Yi B., Zhu L. (2019). A Novel Epigenetic Mechanism of FXR Inhibiting GLP-1 Secretion via MiR-33 and Its Downstream Targets. Biochem. Biophys. Res. Commun..

[9] Płatek T., Polus A., Góralska J., Raźny U., Dziewońska A., Micek A., Dembińska-Kieć A., Solnica B., Malczewska-Malec M. (2021). Epigenetic Regulation of Processes Related to High Level of Fibroblast Growth Factor 21 in Obese Subjects. Genes.

[10] Chandra R., Wang Y., Shahid R.A., Vigna S.R., Freedman N.J., Liddle R.A. (2013). Immunoglobulin-like Domain Containing Receptor 1 Mediates Fat-Stimulated Cholecystokinin Secretion. J. Clin. Investig..

[11] Osinski C., Le Gléau L., Poitou C., de Toro-Martin J., Genser L., Fradet M., Soula H.A., Leturque A., Blugeon C., Jourdren L. (2021). Type 2 Diabetes Is Associated with Impaired Jejunal Enteroendocrine GLP-1 Cell Lineage in Human Obesity. Int. J. Obes..

[12] Reinehr T., Kratzsch J., Kiess W., Andler W. (2005). Circulating Soluble Leptin Receptor, Leptin, and Insulin Resistance before and after Weight Loss in Obese Children. Int. J. Obes..

[13] Cui H., López M., Rahmouni K. (2017). The Cellular and Molecular Bases of Leptin and Ghrelin Resistance in Obesity. Nat. Rev. Endocrinol..

[14] Kamińska A., Kopczyńska E., Bronisz A., Żmudzińska M., Bieliński M., Borkowska A., Tyrakowski T., Junik R. (2010). An Evaluation of Visfatin Levels in Obese Subjects. Endokrynol. Pol..

[15] Taşkesen D., Kirel B., Us T. (2012). Serum Visfatin Levels, Adiposity and Glucose Metabolism in Obese Adolescents. J. Clin. Res. Pediatr. Endocrinol..

[16] Castan-Laurell I., Dray C., Attané C., Duparc T., Knauf C., Valet P. (2011). Apelin, Diabetes, and Obesity. Endocrine.

[17] Zhang X., Yeung D.C.Y., Karpisek M., Stejskal D., Zhou Z.G., Liu F., Wong R.L.C., Chow W.S., Tso A.W.K., Lam K.S.L. (2008). Serum FGF21 Levels Are Increased in Obesity and Are Independently Associated with the Metabolic Syndrome in Humans. Diabetes.

[18] Baek J., Nam H.-K., Rhie Y.-J., Lee K.-H. (2017). Serum FGF21 Levels in Obese Korean Children and Adolescents. J. Obes. Metab. Syndr..

[19] Li G., Yin J., Fu J., Li L., Grant S.F.A., Li C., Li M., Mi J., Li M., Gao S. (2017). FGF21 Deficiency Is Associated with Childhood Obesity, Insulin Resistance and Hypoadiponectinaemia: The BCAMS Study. Diabetes Metab..

[20] Reinehr T., Woelfle J., Roth C.L. (2011). Lack of Association between Apelin, Insulin Resistance, Cardiovascular Risk Factors, and Obesity in Children: A Longitudinal Analysis. Metabolism.

[21] Tapan S., Tascilar E., Abaci A., Sonmez A., Kilic S., Erbil M.K., Ozcan O. (2010). Decreased Plasma Apelin Levels in Pubertal Obese Children. J. Pediatr. Endocrinol. Metab..

[22] Vehapoglu A., Ustabas F., Ozgen T.I., Terzioglu S., Cermik B.B., Ozen O.F. (2015). Role of Circulating Adipocytokines Vaspin, Apelin, and Visfatin in the Loss of Appetite in Underweight Children: A Pilot Trial. J. Pediatr. Endocrinol. Metab..

[23] Weiss R., Dufour S., Groszmann A., Petersen K., Dziura J., Taksali S.E., Shulman G., Caprio S. (2003). Low Adiponectin Levels in Adolescent Obesity: A Marker of Increased Intramyocellular Lipid Accumulation. J. Clin. Endocrinol. Metab..

[24] Araki S., Dobashi K., Kubo K., Asayama K., Shirahata A. (2006). High Molecular Weight, Rather than Total, Adiponectin Levels Better Reflect Metabolic Abnormalities Associated with Childhood Obesity. J. Clin. Endocrinol. Metab..

[25] Yang W.S., Lee W.J., Funahashi T., Tanaka S., Matsuzawa Y., Chao C.L., Chen C.L., Tai T.Y., Chuang L.M. (2002). Plasma Adiponectin Levels in Overweight and Obese Asians. Obes. Res..

[26] Tschöp M., Weyer C., Tataranni P.A., Devanarayan V., Ravussin E., Heiman M.L. (2001). Circulating Ghrelin Levels Are Decreased in Human Obesity. Diabetes.

[27] Hira T., Pinyo J., Hara H. (2020). What Is GLP-1 Really Doing in Obesity?. Trends Endocrinol. Metab..

[28] Gerber M., Boettner A., Seidel B., Lammert A., Bär J., Schuster E., Thiery J., Kiess W., Kratzsch J. (2005). Serum Resistin Levels of Obese and Lean Children and Adolescents: Biochemical Analysis and Clinical Relevance. J. Clin. Endocrinol. Metab..

[29] Lee J.H., Chan J.L., Yiannakouris N., Kontogianni M., Estrada E., Seip R., Orlova C., Mantzoros C.S. (2003). Circulating Resistin Levels Are Not Associated with Obesity or Insulin Resistance in Humans and Are Not Regulated by Fasting or Leptin Administration: Cross-Sectional and Interventional Studies in Normal, Insulin-Resistant, and Diabetic Subjects. J. Clin. Endocrinol. Metab..

[30] Zou C.C., Liang L., Hong F., Fu J.F., Zhao Z.Y. (2005). Serum Adiponectin, Resistin Levels and Non-Alcoholic Fatty Liver Disease in Obese Children. Endocr. J..

[31] Rehfeld J.F. (2020). Measurement of Cholecystokinin in Plasma with Reference to Nutrition Related Obesity Studies. Nutr. Res..

[32] *FTO* Gene Symbol Report | HUGO Gene Nomenclature Committee. https://www.genenames.org/data/gene-symbol-report/#!/hgnc_id/HGNC:24678.

[33] Tweedie S., Braschi B., Gray K., Jones T.E.M., Seal R.L., Yates B., Bruford E.A. (2021). Genenames.Org: The HGNC and VGNC Resources in 2021. Nucleic Acids Res..

[34] Frayling T.M., Timpson N.J., Weedon M.N., Zeggini E., Freathy R.M., Lindgren C.M., Perry J.R.B., Elliott K.S., Lango H., Rayner N.W. (2007). A Common Variant in the *FTO* Gene Is Associated with Body Mass Index and Predisposes to Childhood and Adult Obesity. Science.

[35] Justice A.E., Chittoor G., Blanco E., Graff M., Wang Y., Albala C., Santos J.L., Angel B., Lozoff B., Voruganti V.S. (2019). Genetic Determinants of BMI from Early Childhood to Adolescence: The Santiago Longitudinal Study. Pediatr. Obes..

[36] da Silva T.E.R., Andrade N.L., de Oliveira Cunha D., Leão-Cordeiro J.A.B., Vilanova-Costa C.A.S.T., Silva A.M.T.C. (2018). The *FTO* Rs9939609 Polymorphism and Obesity Risk in Teens: Evidence-Based Meta-Analysis. Obes. Res. Clin. Pract..

[37] Cheng Y., Monteiro C., Matos A., You J., Fraga A., Pereira C., Catalán V., Rodríguez A., Gómez-Ambrosi J., Frühbeck G. (2018). Epigenome-Wide DNA Methylation Profiling of Periprostatic Adipose Tissue in Prostate Cancer Patients with Excess Adiposity-a Pilot Study. Clin. Epigenetics.

[38] Kirchner H., Sinha I., Gao H., Ruby M.A., Schönke M., Lindvall J.M., Barrès R., Krook A., Näslund E., Dahlman-Wright K. (2016). Altered DNA Methylation of Glycolytic and Lipogenic Genes in Liver from Obese and Type 2 Diabetic Patients. Mol. Metab..

[39] Li M., Zou D., Li Z., Gao R., Sang J., Zhang Y., Li R., Xia L., Zhang T., Niu G. (2019). EWAS Atlas: A Curated Knowledgebase of Epigenome-Wide Association Studies. Nucleic Acids Res..

[40] Mehrdad M., Doaei S., Gholamalizadeh M., Fardaei M., Fararouei M., Eftekhari M.H. (2020). Association of *FTO* Rs9939609 Polymorphism with Serum Leptin, Insulin, Adiponectin, and Lipid Profile in Overweight Adults. Adipocyte.

[41] Genis-Mendoza A.D., Martínez-Magaña J.J., Ruiz-Ramos D., Gonzalez-Covarrubias V., Tovilla-Zarate C.A., Narvaez M.L.L., Castro T.B.G., Juárez-Rojop I.E., Nicolini H. (2020). Interaction of *FTO* Rs9939609 and the Native American-Origin ABCA1 p.Arg230Cys with Circulating Leptin Levels in Mexican Adolescents Diagnosed with Eating Disorders: Preliminary Results. Psychiatry Res..

[42] Duicu C., MǍrginean C.O., VoidǍzan S., Tripon F., BǍnescu C. (2016). *FTO* Rs 9939609 SNP Is Associated with Adiponectin and Leptin Levels and the Risk of Obesity in a Cohort of Romanian Children Population. Medicine (Baltimore).

[43] Ghafarian-Alipour F., Ziaee S., Ashoori M.R., Zakeri M.S., Boroumand M.A., Aghamohammadzadeh N., Abbasi-Majdi M., Shool F., Asbaghi N.S., Mohammadi A. (2018). Association between *FTO* Gene Polymorphisms and Type 2 Diabetes Mellitus, Serum Levels of Apelin and Androgen Hormones among Iranian Obese Women. Gene.

[44] Majdi M.A., Mohammadzadeh N.A., Lotfi H., Mahmoudi R., Alipour F.G., Shool F., Moghanloo M.N., Porfaraj S., Zarghami N. (2017). Correlation of Resistin Serum Level with Fat Mass and Obesity-Associated Gene (*FTO*) Rs9939609 Polymorphism in Obese Women with Type 2 Diabetes. Diabetes Metab. Syndr. Clin. Res. Rev..

[45] López-Bermejo A., Petry C.J., Díaz M., Sebastiani G., De Zegher F., Dunger D.B., Ibáñez L. (2008). The Association between the *FTO* Gene and Fat Mass in Humans Develops by the Postnatal Age of Two Weeks. J. Clin. Endocrinol. Metab..

[46] Magno F.C.C.M., Guaraná H.C., Fonseca A.C.P., Cabello G.M.K., Carneiro J.R.I., Pedrosa A.P., Ximenes A.C., Rosado E.L. (2018). Influence of *FTO* Rs9939609 Polymorphism on Appetite, Ghrelin, Leptin, IL6, TNFα Levels, and Food Intake of Women with Morbid Obesity. Diabetes, Metab. Syndr. Obes. Targets Ther..

[47] Karra E., O’Daly O.G., Choudhury A.I., Yousseif A., Millership S., Neary M.T., Scott W.R., Chandarana K., Manning S., Hess M.E. (2013). A Link between *FTO*, Ghrelin, and Impaired Brain Food-Cue Responsivity. J. Clin. Investig..

[48] Dorling J.L., Clayton D.J., Jones J., Carter W.G., Thackray A.E., King J.A., Pucci A., Batterham R.L., Stensel D.J. (2019). A Randomized Crossover Trial Assessing the Effects of Acute Exercise on Appetite, Circulating Ghrelin Concentrations, and Butyrylcholinesterase Activity in Normal-Weight Males with Variants of the Obesity-Linked *FTO* Rs9939609 Polymorphism. Am. J. Clin. Nutr..

[49] *PLAG1* Gene Symbol Report | HUGO Gene Nomenclature Committee. https://www.genenames.org/data/gene-symbol-report/#!/hgnc_id/HGNC:9045.

[50] *PLAG1*—Zinc Finger Protein *PLAG1*—Homo Sapiens (Human)—*PLAG1* Gene & Protein. https://www.uniprot.org/uniprot/Q6DJT9.

[51] Bateman A., Martin M.-J., Orchard S., Magrane M., Agivetova R., Ahmad S., Alpi E., Bowler-Barnett E.H., Britto R., Bursteinas B. (2021). UniProt: The Universal Protein Knowledgebase in 2021. Nucleic Acids Res..

[52] Warrington N.M., Beaumont R.N., Horikoshi M., Day F.R., Helgeland Ø., Laurin C., Bacelis J., Peng S., Hao K., Feenstra B. (2019). Maternal and Fetal Genetic Effects on Birth Weight and Their Relevance to Cardio-Metabolic Risk Factors. Nat. Genet..

[53] Hübel C., Gaspar H.A., Coleman J.R.I., Finucane H., Purves K.L., Hanscombe K.B., Prokopenko I., Graff M., Ngwa J.S., Workalemahu T. (2019). Genomics of Body Fat Percentage May Contribute to Sex Bias in Anorexia Nervosa. Am. J. Med. Genet. Part. B Neuropsychiatr. Genet..

[54] Shungin D., Winkler T., Croteau-Chonka D.C., Ferreira T., Locke A.E., Mägi R., Strawbridge R.J., Pers T.H., Fischer K., Justice A.E. (2015). New Genetic Loci Link Adipose and Insulin Biology to Body Fat Distribution. Nature.

[55] Czogała W., Czogała M., Strojny W., Wątor G., Wołkow P., Wójcik M., Multanowski M.B., Tomasik P., Wędrychowicz A., Kowalczyk W. (2021). Methylation and Expression of *FTO* and *PLAG1* Genes in Childhood Obesity: Insight into Anthropometric Parameters and Glucose-Lipid Metabolism. Nutrients.

[56] Hashimoto K., Kokubun S., Itoi E., Roach H.I. (2007). Improved Quantification of DNA Methylation Using Methylation-Sensitive Restriction Enzymes and Real-Time PCR. Epigenetics.

[57] Redshaw N., Huggett J.F., Taylor M.S., Foy C.A., Devonshire A.S. (2014). Quantification of Epigenetic Biomarkers: An Evaluation of Established and Emerging Methods for DNA Methylation Analysis. BMC Genom..

[58] Howe K.L., Achuthan P., Allen J., Allen J., Alvarez-Jarreta J., Ridwan Amode M., Armean I.M., Azov A.G., Bennett R., Bhai J. (2021). Ensembl 2021. Nucleic Acids Res..

[59] Untergasser A., Cutcutache I., Koressaar T., Ye J., Faircloth B.C., Remm M., Rozen S.G. (2012). Primer3—New Capabilities and Interfaces. Nucleic Acids Res..

[60] R: The R Project for Statistical Computing. https://www.r-project.org/.

[61] Smyth G.K. (2004). Linear Models and Empirical Bayes Methods for Assessing Differential Expression in Microarray Experiments. Stat. Appl. Genet. Mol. Biol..

[62] Gentleman R.C., Carey V.J., Bates D.M., Bolstad B., Dettling M., Dudoit S., Ellis B., Gautier L., Ge Y., Gentry J. (2004). Bioconductor: Open Software Development for Computational Biology and Bioinformatics. Genome Biol..

[63] Benjamini Y., Hochberg Y. (1995). Controlling the False Discovery Rate: A Practical and Powerful Approach to Multiple Testing. J. R. Stat. Soc. Ser. B.

[64] WHO Anthro Survey Analyser and Other Tools. https://www.who.int/toolkits/child-growth-standards/software.

[65] *FTO* Gene—GeneCards | *FTO* Protein | *FTO*. Antibody. https://www.genecards.org/cgi-bin/carddisp.pl?gene=FTO&keywords=FTO.

[66] Stelzer G., Rosen N., Plaschkes I., Zimmerman S., Twik M., Fishilevich S., Stein T.I., Nudel R., Lieder I., Mazor Y. (2016). The GeneCards Suite: From Gene Data Mining to Disease Genome Sequence Analyses. Curr. Protoc. Bioinform..

[67] O’Leary N.A., Wright M.W., Brister J.R., Ciufo S., Haddad D., McVeigh R., Rajput B., Robbertse B., Smith-White B., Ako-Adjei D. (2016). Reference Sequence (RefSeq) Database at NCBI: Current Status, Taxonomic Expansion, and Functional Annotation. Nucleic Acids Res..

[68] Jia G., Yang C.G., Yang S., Jian X., Yi C., Zhou Z., He C. (2008). Oxidative Demethylation of 3-Methylthymine and 3-Methyluracil in Single-Stranded DNA and RNA by Mouse and Human *FTO*. FEBS Lett..

[69] Han Z., Niu T., Chang J., Lei X., Zhao M., Wang Q., Cheng W., Wang J., Feng Y., Chai J. (2010). Crystal Structure of the *FTO* Protein Reveals Basis for Its Substrate Specificity. Nature.

[70] Speakman J.R. (2015). The ‘Fat Mass and Obesity Related’ (*FTO*) Gene: Mechanisms of Impact on Obesity and Energy Balance. Curr. Obes. Rep..

[71] Di Renzo L., Cioccoloni G., Falco S., Abenavoli L., Moia A., Sinibaldi Salimei P., De Lorenzo A. (2018). Influence of *FTO* Rs9939609 and Mediterranean Diet on Body Composition and Weight Loss: A Randomized Clinical Trial NCT01890070 NCT. J. Transl. Med..

[72] Chang J.Y., Park J.H., Park S.E., Shon J., Park Y.J. (2018). The Fat Mass- and Obesity-Associated (*FTO*) Gene to Obesity: Lessons from Mouse Models. Obesity.

[73] Labayen I., Ruiz J.R., Ortega F.B., Dalongeville J., Jiménez-Pavón D., Castillo M.J., De Henauw S., González-Gross M., Bueno G., Molnar D. (2011). Association between the *FTO* Rs9939609 Polymorphism and Leptin in European Adolescents: A Possible Link with Energy Balance Control. The HELENA Study. Int. J. Obes..

[74] Huang Z., Luo X., Liu M., Chen L. (2019). Function and Regulation of Apelin/APJ System in Digestive Physiology and Pathology. J. Cell Physiol..

[75] Kadoglou N.P.E., Vrabas I.S., Kapelouzou A., Lampropoulos S., Sailer N., Kostakis A., Liapis C.D., Angelopoulou N. (2012). The Impact of Aerobic Exercise Training on Novel Adipokines, Apelin and Ghrelin, in Patients with Type 2 Diabetes. Med. Sci. Monit..

[76] Son J.S., Chae S.A., Park B.I., Du M., Song W. (2019). Plasma Apelin Levels in Overweight/Obese Adults Following a Single Bout of Exhaustive Exercise: A Preliminary Cross-Sectional Study. Endocrinol. Diabetes Nutr..

[77] Kadoglou N.P.E., Vrabas I.S., Kapelouzou A., Angelopoulou N. (2012). The Association of Physical Activity with Novel Adipokines in Patients with Type 2 Diabetes. Eur. J. Intern. Med..

[78] Chandra R., Liddle R.A. (2007). Cholecystokinin. Curr. Opin. Endocrinol. Diabetes Obes..

[79] Crovesy L., Rosado E.L. (2019). Interaction between Genes Involved in Energy Intake Regulation and Diet in Obesity. Nutrition.

[80] Holst J.J. (2007). The Physiology of Glucagon-like Peptide 1. Physiol. Rev..

[81] Danaher J., Stathis C.G., Cooke M.B. (2019). Similarities in Metabolic Flexibility and Hunger Hormone Ghrelin Exist between *FTO* Gene Variants in Response to an Acute Dietary Challenge. Nutrients.

[82] Tanaka T., Ngwa J.S., Van Rooij F.J.A., Zillikens M.C., Wojczynski M.K., Frazier-Wood A.C., Houston D.K., Kanoni S., Lemaitre R.N., Luan J.N.A. (2013). Genome-Wide Meta-Analysis of Observational Studies Shows Common Genetic Variants Associated with Macronutrient Intake. Am. J. Clin. Nutr..

[83] *PLAG1* Gene—GeneCards | *PLAG1* Protein | *PLAG1* Antibody. https://www.genecards.org/cgi-bin/carddisp.pl?gene=PLAG1&keywords=PLAG1.

[84] Van Dyck F., Declercq J., Braem C., Van de Ven W. (2007). *PLAG1*, the Prototype of the PLAG Gene Family: Versatility in Tumour Development (Review). Int. J. Oncol..

[85] Zatkova A., Rouillard J.M., Hartmann W., Lamb B.J., Kuick R., Eckart M., Von Schweinitz D., Koch A., Fonatsch C., Pietsch T. (2004). Amplification and Overexpression of the IGF2 Regulator *PLAG1* in Hepatoblastoma. Genes Chromosom. Cancer.

[86] GWAS Catalog. https://www.ebi.ac.uk/gwas/home.

[87] Buniello A., Macarthur J.A.L., Cerezo M., Harris L.W., Hayhurst J., Malangone C., McMahon A., Morales J., Mountjoy E., Sollis E. (2019). The NHGRI-EBI GWAS Catalog of Published Genome-Wide Association Studies, Targeted Arrays and Summary Statistics 2019. Nucleic Acids Res..

[88] Street M.E., Smerieri A., Montanini L., Predieri B., Iughetti L., Valenzise M., De Luca F., Vigone M., Weber G., Maghnie M. (2013). Interactions among Pro-Inflammatory Cytokines, IGF System and Thyroid Function in Pre-Pubertal Obese Subjects. J. Biol. Regul. Homeost. Agents.

[89] Seck T., Englaro P., Blum W.F., Scheidt-Nave C., Rascher W., Ziegler R., Pfeilschifter J. (1998). Leptin Concentrations in Serum from a Randomly Recruited Sample of 50- to 80-Year-Old Men and Women: Positive Association with Plasma Insulin-like Growth Factors (IGFs) and IGF-Binding Protein-3 in Lean, but Not in Obese, Individuals. Eur. J. Endocrinol..

[90] Kleiman A., Keats E.C., Chan N.G., Khan Z.A. (2013). Elevated IGF2 Prevents Leptin Induction and Terminal Adipocyte Differentiation in Hemangioma Stem Cells. Exp. Mol. Pathol..

[91] Uchida A., Zechner J.F., Mani B.K., Park W.M., Aguirre V., Zigman J.M. (2014). Altered Ghrelin Secretion in Mice in Response to Diet-Induced Obesity and Roux-En-Y Gastric Bypass. Mol. Metab..

[92] de Mello V.D.F., Kolehmanien M., Schwab U., Pulkkinen L., Uusitupa M. (2012). Gene Expression of Peripheral Blood Mononuclear Cells as a Tool in Dietary Intervention Studies: What Do We Know so Far?. Mol. Nutr. Food Res..

[93] Reynés B., Priego T., Cifre M., Oliver P., Palou A. (2018). Peripheral Blood Cells, a Transcriptomic Tool in Nutrigenomic and Obesity Studies: Current State of the Art. Compr. Rev. Food Sci. Food Saf..

[94] Kohane I.S., Valtchinov V.I. (2012). Quantifying the white blood cell transcriptome as an accessible window to the multiorgan transcriptome. Bioinformatics.

