# Supplementary material for: FTO and PLAG1 Genes Expression and FTO Methylation Predict Changes in Circulating Levels of Adipokines and Gastrointestinal Peptides in Children
Source: Nutrients. 2021 Oct 13;13(10):3585. doi: 10.3390/nu13103585 (PMC8538237; doi:10.3390/nu13103585)
Supplement: Supplementary file 1 [file nutrients-13-03585-s001.zip › nutrients-1374005-supplementary.pdf]

*nutrients*

MDPI

*Article*

# *FTO* and *PLAG1* Genes Expression and *FTO* Methylation Predict Changes in Circulating Levels of Adipokines and Gastrointestinal Peptides in Children

**Wojciech Czogała [1,5], Wojciech Strojny [1], Magdalena Schab [1], Agnieszka Grabowska [2], Karol Miklusiak [3], Wojciech Kowalczyk [3], Agnieszka Łazarczyk [3], Przemysław Tomasik [4] and Szymon Skoczeń [1,5,*]**

## Supplementary

Methylation regions, based on Methyl-DIP (Nimblegene), tissue Whole Blood (source: Ensembl).

FTO (Chr16)

Region 1 (FTO – upstream)

53703684-53703899

53703663 ACGCCAGCAGAACTCCAGGGCCAACTCCAGGGCCTTCTCCAGGCGGCAGAGCGGA CCCTA 53703722
53703723 GGACC**CCGG**CCCGCGCTGCAGTGGGGAGGGTCAGCAACCTCCACCCACCCTCATCCTCCC 53703782
53703783 CCATCCTC**CCGG**GTACTCACCGTGCCACTGGCCCT GCAGCTAGCTACCGTTGCTATAGCG 53703842
53703843 CCGACAGCGTGGCGGGCGGCTGGCCGAGAGGAGCACGGGAGAAACATGGCAGGCTCCCGT 53703902

PLAG1 (Chr8)

Region 1 (PLAG1 exon1)

56211059-56211208

56211264 **ACAATGGCTGCTGGAAAGAGGCGTAAGGAAACAATTTCCAGGCCCGCCGCGTCCAGCCCG** 56211205
56211204 **AAATA TGAGAAAAAAATTATTAGAAATTCCGCGGGCGGTGTAGAGGCGGCGGACGGGCCG** 56211145
56211144 **GAGGGAGGATGTTAAAGCCCCGCG**GTGAGTTCT**CCGG**GGGT**CCGG**GGCGGCGGCGAGGCG 56211085
56211084 TTTAGCGGGAGAAATATCAGGGTTAT TTAAATTATGGGACTAGCCGAGGGGGCAGAGGAG 56211025

**Table S1.** The metabolic parameters of the study group (including values at OGTT time points). Values are presented as mean ± standard deviation.

| Baseline characteristics | Study group N = 26 |
|---|---|
| Adiponectin -OGTT 0 min (μg/ml) | 3.47 ± 2.2 |
| Adiponectin – OGTT 60 min (μg/ml) | 3.42 ± 2.2 |
| Adiponectin – OGTT 120 min (μg/ml) | 3.4 ± 2.24 |
| Apelin – OGTT 0 min (ng/ml) | 2.62 ± 1.83 |
| Apelin- OGTT 60 min (ng/ml) | 2.43 ± 1.72 |
| Apelin – OGTT 120 min (ng/ml) | 2.44 ± 1.83 |
| Leptin -OGTT 0 min (ng/ml) | 28.03 ± 26.33 |
| Leptin- OGTT 60 min (ng/ml) | 27.99 ± 24.85 |
| Leptin – OGTT 120 min (ng/ml) | 26.15 ± 25.4 |
| Leptin receptor – OGTT 0 min (ng/ml) | 10.56 ± 6.64 |
| Leptin receptor – OGTT 60 min (ng/ml) | 10.82 ± 6.88 |
| Leptin receptor – OGTT 120 min (ng/ml) | 10.56 ± 6.73 |
| Resistin – OGTT 0 min (ng/ml) | 3.76 ± 1.26 |
| Resistin- OGTT 60 min (ng/ml) | 3.74 ± 1.05 |
| Resistin – OGTT 120 min (ng/ml) | 3.71 ± 0.91 |
| Visfatin- OGTT 0 min | 11.53 ± 4.92 |
| Visfatin- OGTT 60 min | 9.69 ± 5.26 |
| Visfatin-OGTT 120 min | 9.99 ± 5.49 |
| FGF21- OGTT 0 min (ng/ml) | 139.74 ± 91.06 |
| FGF21- OGTT 60 min (ng/ml) | 114.63 ± 67.89 |
| FGF21-OGTT 120 min (ng/ml) | 160.49 ± 106.76 |
| Cholecystokinin- OGTT 0 min (ng/ml) | 2.5 ± 1.45 |
| Cholecystokinin – OGTT 60 min (ng/ml) | 2.02 ± 1.39 |
| Cholecystokinin- OGTT 120 min (ng/ml) | 2.69 ± 1.61 |
| Ghrelin- OGTT 0 min (ng/ml) | 497.86 ± 153.34 |
| Ghrelin- OGTT 60 min (ng/ml) | 461.82 ± 144.66 |
| Ghrelin – OGTT 120 min (ng/ml) | 470.4 ± 156.3 |
| GLP-1 – OGTT 0 min (ng/ml) | 1.03 ± 0.5 |
| GLP-1 – OGTT 60 min (ng/ml) | 0.88 ± 0.4 |
| GLP-1 -OGTT 120 min (ng/ml) | 0.91 ± 0.2 |

**Table S2.** Correlation results of adipokines serum levels with *FTO* gene methylation, expression and *PLAG1* gene expression (including values at OGTT time points).

| Adipokines | *FTO* gene methylation | | *FTO* expression | | *PLAG1* expression | |
|---|---|---|---|---|---|---|
| | Spearman's correlation coefficient r | p/p$^{PH}$ value | Spearman's correlation coefficient r | p/ p$^{PH}$ value | Spearman's correlation coefficient r | p/ p$^{PH}$ value |
| Adiponectin- OGTT 0 min (μg/ml) | −0.307 | 0.144/0.288 | −0.104 | 0.63/0.63 | 0.19 | 0.375/0.45 |
| Adiponectin – OGTT 60 min (μg/ml) | −0.214 | 0.327/0.374 | −0.2 | 0.36/0.393 | −0.112 | 0.612/0.668 |
| Adiponectin- OGTT 120 min (μg/ml) | −0.360 | 0.100/0.3. | −0.189 | 0.399/0.416 | 0.029 | 0.897/0.936 |
| Apelin – OGTT 0 min (ng/ml) | −0.301 | 0.163/0.279 | −0.808 | **<0.001/<0.001** | −0.63 | **0.001/006** |
| Apelin – OGTT 60 min (ng/ml) | −0.398 | 0.059/0.354. | −0.758 | **<0.001/<0.001** | −0.622 | **0.002/0.008** |
| Apelin- OGTT 120 min (ng/ml) | −0.341 | 0.111/0.242. | −0.703 | **<0.001/<0.001** | −0.509 | **0.013/0.039** |
| Leptin – OGTT 0 min (ng/ml) | 0.292 | 0.166/0.249 | 0.756 | **<0.001/<0.001** | 0.707 | **<0.001/0.001** |
| Leptin – OGTT 60 min (ng/ml) | 0.276 | 0.191/0.270 | 0.718 | **<0.001/<0.001** | 0.708 | **<0.001/0.003** |
| Leptin – OGTT 120 min (ng/ml) | 0.338 | 0.105/0.280. | 0.698 | **<0.001/<0.001** | 0.601 | **0.002/0.010** |
| Leptin receptor – OGTT 0 min (ng/ml) | −0.303 | 0.151/0.279 | −0.621 | **0.001/<0.001** | −0.45 | **0.027**/0.054 |
| Leptin receptor – 60 min (ng/ml) | −0.231 | 0.278/0.351 | −0.571 | **0.004/0.009** | −0.467 | **0.021/0.046** |
| Leptin receptor – OGTT 120 min (ng/ml) | −0.081 | 0.713/0.731 | −0.577 | **0.004/0.008** | −0.488 | **0.018/0.043** |
| Resistin – OGTT 0 min (ng/ml) | −0.231 | 0.278/0.344 | −0.314 | 0.134/0.169 | −0.216 | 0.31/0.438 |
| Resistin- OGTT 60 min (ng/ml) | −0.437 | **0.033**/0.264. | −0.507 | **0.011/0.020** | −0.199 | 0.352/0.469 |
| Resistin – OGTT 120 min (ng/ml) | −0.194 | 0.365/ | −0.419 | **0.042**/0.063 | −0.352 | 0.092/0.170 |
| Visfatin – OGTT 0 min (ng/ml) | −0.501 | **0.013**/0.156. | −0.394 | 0.056/0.075 | −0.272 | 0.198/0.317 |
| Visfatin- OGTT 60 min (ng/ml) | −0.523 | **0.011**/0.264. | −0.452 | **0.03**/0.051 | −0.338 | 0.115/0.197 |
| Visfatin – OGTT 120 min (ng/ml) | −0.387 | 0.075/0.3. | −0.31 | 0.16/0.192 | −0.118 | 0.601/0.687 |

**Table S3.** Correlation results of gastrointestinal tract hormones serum levels with FTO gene methylation, expression and PLAG1 gene expression (including values at OGTT time points).

| Gastrointestinal tract hormones | *FTO* gene methylation | | *FTO* expression | | *PLAG1* expression | |
|---|---|---|---|---|---|---|
| | Spearman's correlation coefficient r | p/$p^{BH}$ value | Spearman's correlation coefficient r | p/ $p^{BH}$ value | Spearman's correlation coefficient r | p/ $p^{BH}$ value |
| CCK- OGTT 0 min (ng/ml) | −0.496 | **0.014/0.037** | −0.652 | **0.001/0.016** | −0.507 | **0.012/0.**192 |
| CCK – OGTT 60 min (pg/ml) (ng/ml) | −0.501 | **0.013/0.042** | −0.548 | **0.006/0.014** | −0.365 | 0.079/0.115 |
| CCK – OGTT 120 min (ng/ml) | −0.489 | **0.015/0.034** | −0.513 | **0.011/0.02** | −0.438 | **0.032/**0.128 |
| FGF21- OGTT 0 min (ng/ml) | 0.582 | **0.003/0.048** | 0.498 | **0.013/0.021** | 0.259 | 0.221/0.272 |
| FGF21- OGTT 60 min (ng/ml) | 0.443 | **0.030/**0.06 | 0.436 | **0.033/0.048** | 0.174 | 0.415/0.415 |
| FGF21-OGTT 120 min (ng/ml) | 0.524 | **0.009/0.048** | 0.418 | **0.042/**0.056 | 0.232 | 0.276/0.294 |
| Ghrelin- OGTT 0 min (pg/ml) | −0.238 | 0.262/0.262 | −0.34 | 0.105/0.129 | −0.402 | 0.051/0.117 |
| Ghrelin – OGTT 60 min (ng/ml) | −0.316 | 0.142/0.175 | −0.194 | 0.376/0.401 | −0.396 | 0.061/0.108 |
| Ghrelin – OGTT 120 min (ng/ml) | −0.281 | 0.194/0.207 | −0.146 | 0.505/0.505 | −0.36 | 0.091/0.121 |
| GLP-1 – OGTT 0 min (ng/ml) | −0.347 | 0.105/0.14 | −0.645 | **0.001/0.005** | −0.428 | **0.041/**0.109 |
| GLP-1 – OGTT 60 min (ng/ml) | −0.278 | 0.188/0.215 | −0.652 | **0.001/0.004** | −0.383 | 0.065/0.104 |
| GLP-1 - OGTT 120 min (ng/ml) | −0.398 | 0.054/0.096 | −0.619 | **0.001/0.003** | −0.394 | 0.057/0.114 |
